# Evaluating a Parainfluenza Virus 5-Based Vaccine in a Host with Pre-Existing Immunity against Parainfluenza Virus 5

**DOI:** 10.1371/journal.pone.0050144

**Published:** 2012-11-20

**Authors:** Zhenhai Chen, Pei Xu, Gregory W. Salyards, Stephen B. Harvey, Balazs Rada, Zhen F. Fu, Biao He

**Affiliations:** 1 Department of Infectious Diseases, University of Georgia College of Veterinary Medicine, Athens, Georgia, United States of America; 2 Intercollege Graduate Program in Cell and Developmental Biology, The Pennsylvania State University, University Park, Pennsylvania, United States of America; 3 University Research Animal Resources and the Department of Population Health, College of Veterinary Medicine, University of Georgia, Athens, Georgia, United States of America; 4 Department of Pathology, University of Georgia College of Veterinary Medicine, Athens, Georgia, United States of America; Aaron Diamond AIDS Research Center with the Rockefeller University, United States of America

## Abstract

Parainfluenza virus 5 (PIV5), formerly known as simian virus 5 (SV5), is a paramyxovirus often referred to as canine parainfluenza virus (CPI) in the veterinary field. PIV5 is thought to be a contributing factor to kennel cough. Kennel cough vaccines containing live PIV5 have been used in dogs for many decades. PIV5 is not known to cause any diseases in humans or other animals. PIV5 has been used as a vector for vaccine development for humans and animals. One critical question concerning the use of PIV5 as a vector is whether prior exposure to PIV5 would prevent the use of PIV5-based vaccines. In this work, we have examined immunogenicity of a recombinant PIV5 expressing hemagglutinin (HA) of influenza A virus subtype 3 (rPIV5-H3) in dogs that were immunized against PIV5. We found that vaccination of the dogs containing neutralizing antibodies against PIV5 with rPIV5-H3 generated immunity against influenza A virus, indicting that PIV5-based vaccine is immunogenic in dogs with prior exposure. Furthermore, we have examined exposure of PIV5 in human populations. We have detected neutralizing antibody (nAb) against PIV5 in 13 out of 45 human serum samples (about 29 percent). The nAb titers in humans were lower than that in vaccinated dogs, suggesting that nAb in humans is unlikely to prevent PIV5 from being an efficacious vector in humans.

## Introduction

Parainfluenza Virus 5 (PIV5) is a non-segmented negative strand RNA virus (NNSV). It is a member of the *Rubulavirus* genus of the family *Paramyxoviridae*, which includes mumps virus, human parainfluenza virus type 2 (HPIV2) and type 4 (HPIV4) [1]. The origin and natural host of PIV5 is not clear. PIV5 was first isolated from monkey cells as a contaminant in 1956, hence the original name SV5 [2]. However, subsequent serological testing of wild monkeys indicated no exposure to this virus. In contrast, monkeys in captivity at an animal facility rapidly sero-converted, suggesting they contacted the virus in captivity [3], [4]. All evidence to date indicates that PIV5 is not a simian virus. There is no convincing evidence that PIV5 causes diseases in humans, despite completely unfounded speculation in the 1970’s that PIV5 might be associated with a number of illnesses including multiple sclerosis (MS), subacute sclerosing panencepalitis (SSPE), Creutzfeldt-Jakob disease (CJD), pemphigus, athero-sclerosis, Paget’s disease, hepatitis and the common cold. Subsequent studies have ruled out PIV5 as the etiological agent for any of these diseases [5], [6], [7]. The virus was renamed parainfluenza virus 5 (PIV5) by International Committee on Taxonomy of Viruses in 2009.

**Figure 1:**
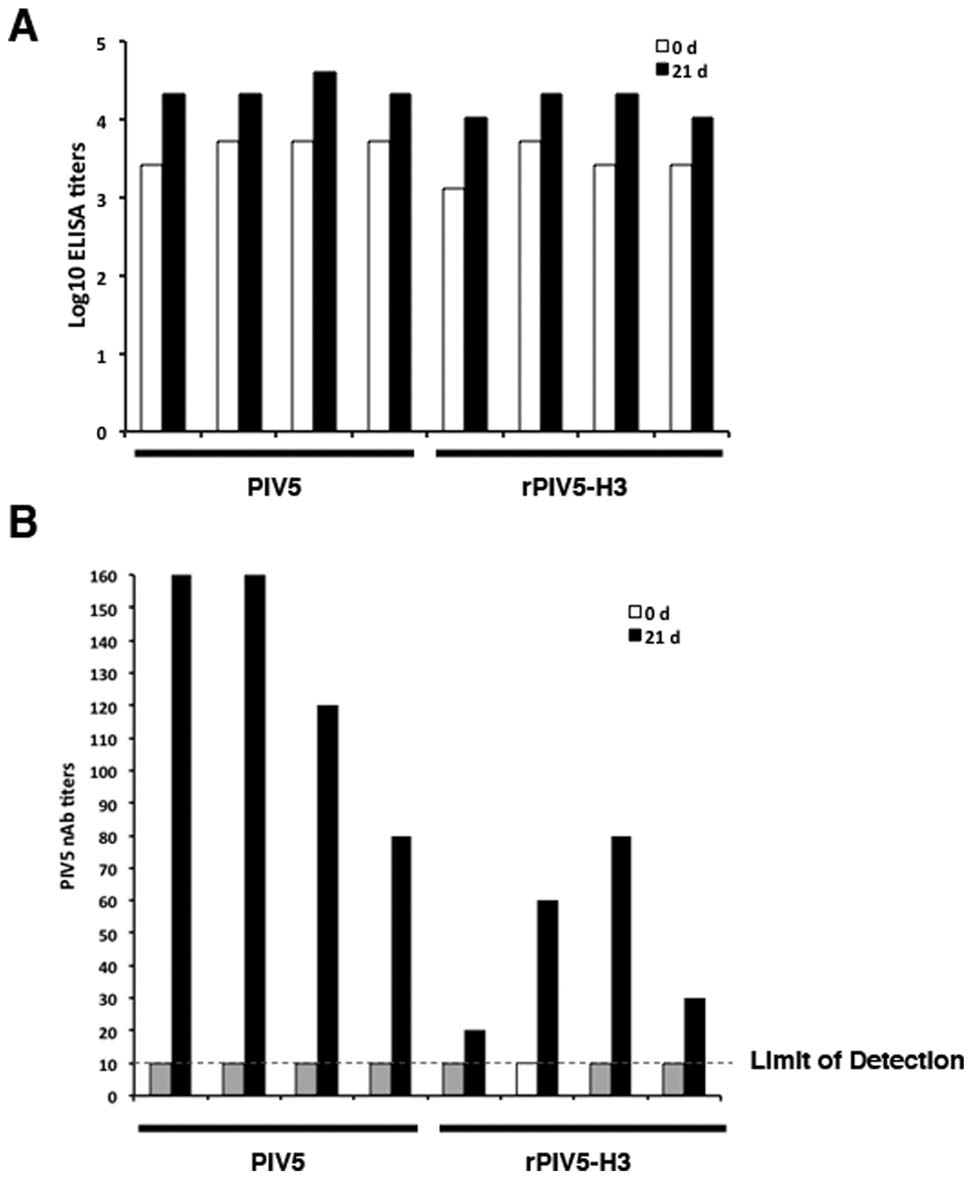
Titers of anti-PIV5 antibodies in dogs without PIV5 exposure. Eight PIV5 naïve dogs were immunized with one dose of 8×10^7^ PFU of PIV5 or rPIV5-H3 viruses by intranasal route. The dogs were divided into two groups: PIV5-infected dogs and rPIV5-H3-infected dogs. Blood samples were collected at 0 and 21 days post infection for ELISA (A) and virus neutralization antibody (nAb) assay (B). The grey columns indicate that the PIV5 nAb titer is less than 10, the limit of detection in this assay. The black columns indicate that the nAb titer is equal to or higher than 10.

The PIV5, a negative non-segmented single-stranded RNA virus (NNSV), is a good viral vector candidate for vaccine development because it does not have a DNA phase in its life cycle, and thus the possible unintended consequences of genetic modifications of host cell DNA through recombination or insertion are avoided. In comparison to positive strand RNA viruses, the genome structure of PIV5 is stable. A recombinant PIV5 expressing green fluorescence protein (GFP) has been generated and the GFP gene was maintained for more than 10 generations (the duration of the experiment) [8]. Thus, PIV5 is better suited as a vaccine vector than positive strand RNA viruses since the genomes of positive strand RNA viruses recombine and often delete the inserted foreign genes quickly [9]. PIV5 infects a large range of cell types including primary human cells as well as established human cell lines [1], [10] and, in spite of extensive testing, we have not found a cell line that is resistant to PIV5 infection. Yet, PIV5 has very little cytopathic effect (CPE) on most infected cells [11], [12]. PIV5 also infects a large number of mammals without being associated with any diseases except kennel cough in dogs [13], [14], [15], [16], [17]. PIV5 can be grown in MDBK cells for more than 40 days as well as in Vero cells, a WHO-approved cell line for vaccine production, for high titers and is released in the media at a titer up to 8×10^8^ PFU/ml, indicating its potential as a cost-effective and safe vaccine vector that may be used in mass production.

It is believed that PIV5 may contribute to kennel cough in dogs [13], [14], [15], [16], [17]. Even though infection of dogs with PIV5 did not lead to kennel cough [18], [19], kennel cough vaccines containing live attenuated PIV5 have been used on dogs over 30 years. Dogs are vaccinated intranasally and dogs often sneeze during the vaccination, exposing veterinary workers and owners as well. The wide use of kennel cough vaccines that contain live PIV5 suggests that PIV5 may be a safe vaccine in humans. In our studies, we have found that a single dose inoculation of recombinant PIV5 expressing hemagglutinin (HA) of subtype 3 (H3) protected against influenza virus challenge in mice [20] and a single dose vaccination as low as 1,000 plaque forming units (PFUs) of a recombinant PIV5 expressing HA of H5N1 protected lethal challenge by highly pathogenic avian influenza virus H5N1 in mice [21].

**Figure 2:**
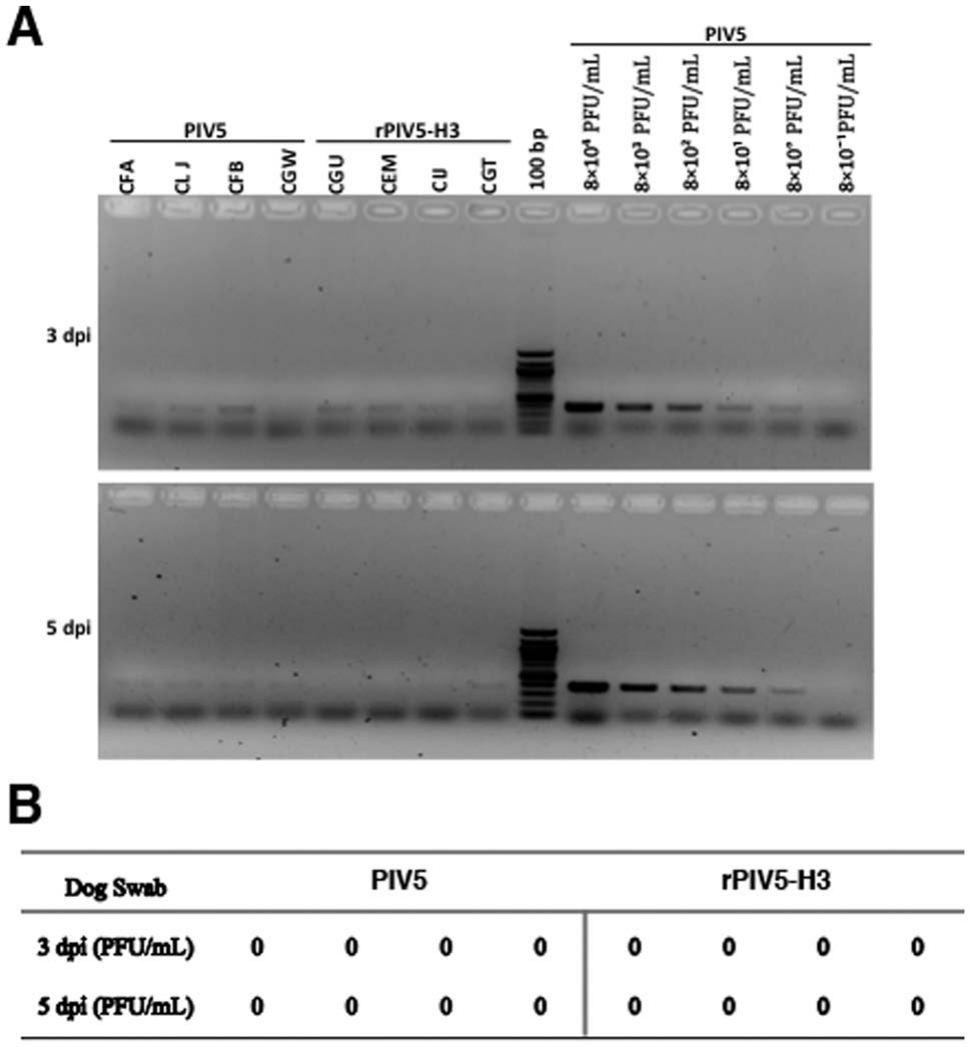
Replication of PIV5 in dogs without PIV5 exposure. The nasal swabs of dogs were collected at 3 and 5 days post infection, and placed into a vial containing 0.5 mL of DMEM with 2% FBS. (A) Detection of virus with RT-PCR. (B) Detection of virus with plaque assay. Swab samples were examined by plaque assay on BHK21 cells. Two replicates for each serially diluted swab sample (1∶10^0^ to 1∶10^2^) were used in the assay.

One critical question concerning the use of PIV5 as a vector is whether prior exposure to PIV5 would prevent the use of PIV5-based vaccine. In this work, we have examined efficacy of a recombinant PIV5 expressing HA (PIV5-HA) of influenza virus in dogs that were immunized against PIV5. Furthermore, we have examined exposure of PIV5 in humans.

## Materials and Methods

### Virus and Cells

MDBK, BHK21 and Vero cells were grown in Dulbecco's modified Eagle medium (DMEM) (Invitrogen) containing 10% fetal bovine serum (FBS) and 100 IU/ml penicillin–100 µg/ml streptomycin. The rPIV5-H3 virus was constructed in a previous report [20], which contains influenza A virus (A/Udorn/72, H3N2 subtype) hemagglutinin (HA) gene. The PIV5 viruses were grown in MDBK cells for 4 to 5 days using DMEM containing 2% FBS and the virus titers were examined by plaque assay on BHK21 cells as previously reported [8]. Briefly, the BHK21 cells in 6-well plates were infected with serially diluted virus (1∶10^1^ to 1∶10^7^). After 2 hours (h), the inoculating mixture was removed and replaced with 4 ml DMEM containing 2% FBS, 100 IU/ml penicillin, 100 µg/ml streptomycin, and 1% low-melting-point agarose. The plaques were counted at 4 to 6 days post infection (dpi). Two replicates for each time point were set for titer calculation. The mumps virus, Jeryl Lynn (JL) vaccine strain, was grown in Vero cells and was harvested at 4 to 7 dpi. Virus titer was measured in Vero cells by plaque assay as described previously [22]. The influenza A/Udorn/72 virus was grown in eggs [23].

**Figure 3:**
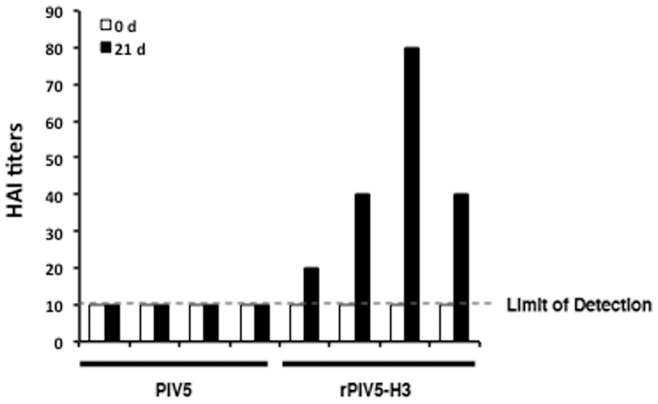
Immune responses in the “PIV5 naïve” dogs inoculated with rPIV5-H3. The dog blood samples were collected at 0 and 21 days post infection. 4 HAU of the influenza A virus (A/Udorn/72, H3N2 subtype) were mixed with serially diluted dog sera in 96-well round-bottom plates. The hemagglutination inhibition (HAI) titer was scored as the reciprocal of the highest dilution antiserum that completely inhibits hemagglutination. The graph shows the mean value of duplicate wells for each dog. The limit of detection of the HAI titer (10) is indicated.

To purify the PIV5 and mumps virus for ELISA assay, viruses in the cleared supernatant were pelleted in a Thermo Scientific ultracentrifuge Type F40L-8×100 rotor at 37,000 rpm for 1 h. The pellets were then resuspended in TNE buffer (10 mM Tris [pH 7.4], 100 mM NaCl, 1 mM EDTA) and loaded onto 10% to 80% sucrose gradient and centrifuged in a TH-641 rotor for 1 h at 37,000 rpm. The virus bands were collected and pelleted in a F40L-8×100 rotor for 1 h at 37,000 rpm. The purified viruses were resuspended in phosphate buffered saline (PBS) buffer (pH 7.4).

**Figure 4:**
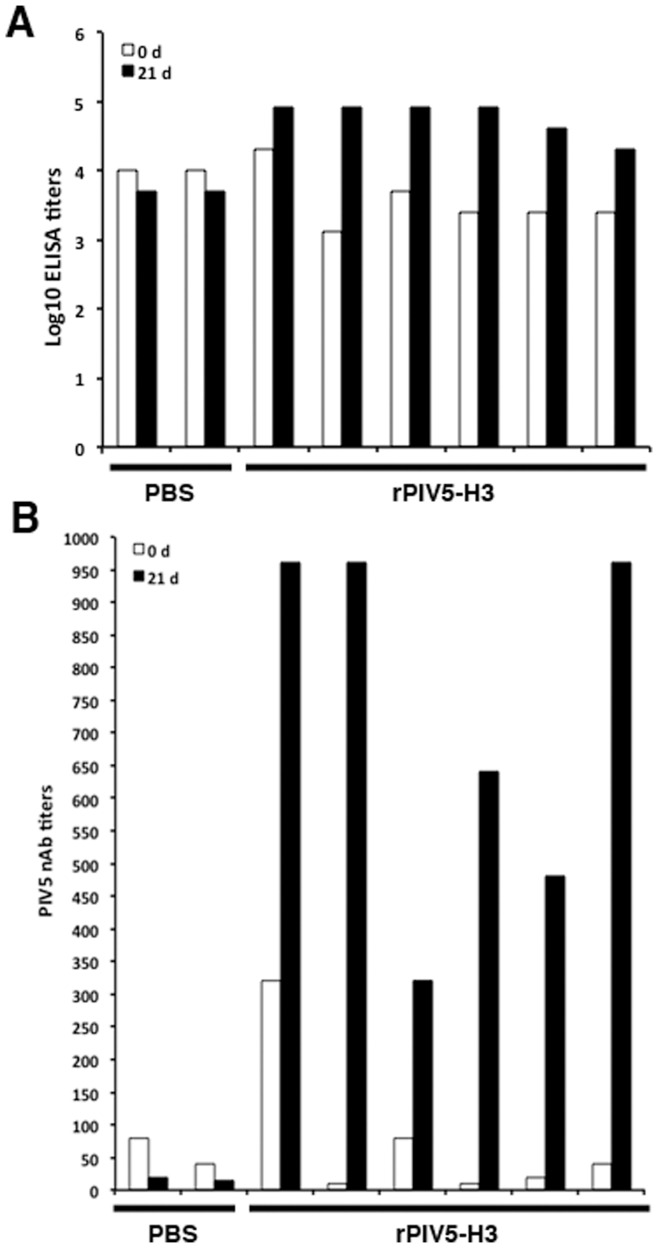
Titers of anti-PIV5 antibodies in the PIV5-vaccinated dogs. Eight dogs which had been vaccinated with live PIV5 were immunized with one dose of 8×10^7^ PFU of rPIV5-H3 viruses in 1 mL or PBS via intranasal route. The dogs were divided into two groups: two dogs received PBS; The remaining six dogs received rPIV5-H3. Blood samples were collected at 0 and 21 days post infection for ELISA (A) and viral neutralization antibody assay (B). Data were presented as average value of duplicate wells. In the neutralization antibody assay, the white column indicates the PIV5 nAb titer is equal to or higher than 10.

### Ethic Statement about Animal Use

This study was carried out in strict accordance with the recommendations in the Guide for the Care and Use of Laboratory Animals of the National Institutes of Health. The protocol was approved by the Committee on the Ethics of Animal Experiments of the University of Georgia (Permit Number: A2011 12-012-Y1-A3). All efforts were made to minimize suffering. Dogs used in this study were housed and cared for in accordance with The Guide for the Care and Use of Laboratory Animals (eighth edition).

**Figure 5:**
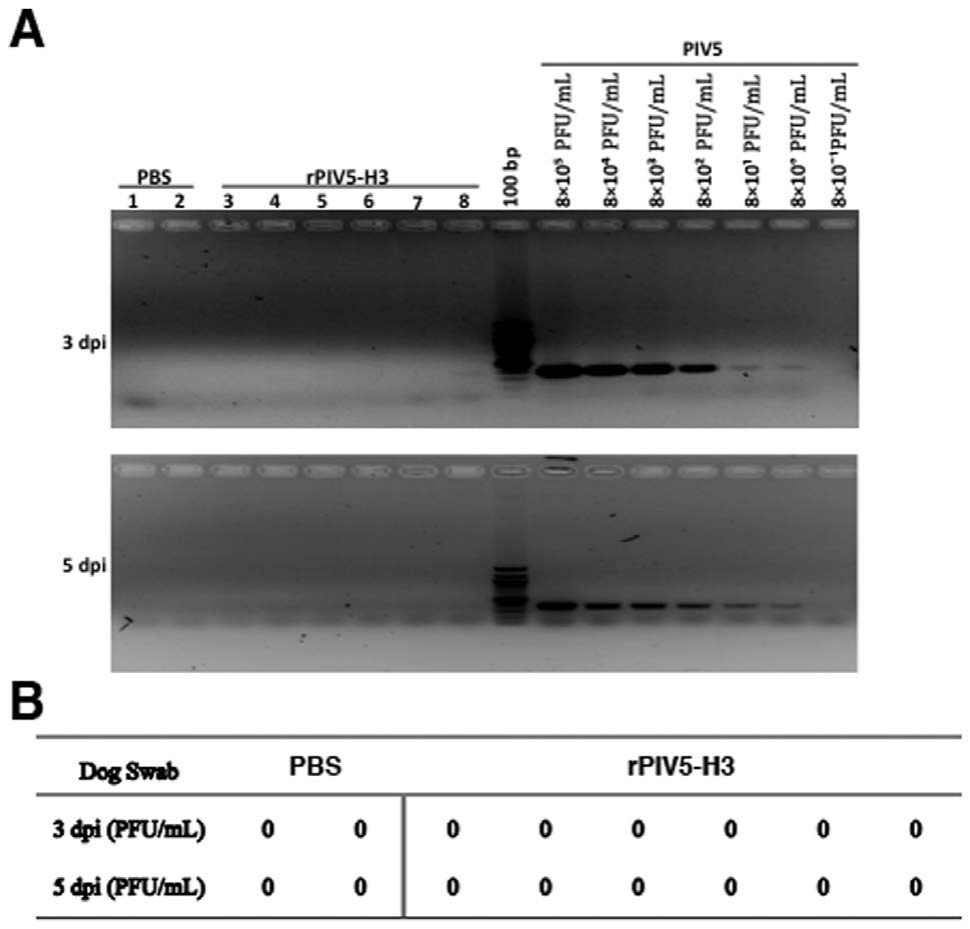
Replication of PIV5 in dogs with prior PIV5 vaccination. The nasal swabs of dogs were collected at 3 and 5 dpi. Detections of virus were performed the same as in Fig. 2. (A) RT-PCR and (B) Plaque assay.

### Infection of Dogs with PIV5 or rPIV5-H3

Purpose-bred dogs utilized in this study were purchased from Covance Research Products (482 Frenchs Store Road, Cumberland, VA). In the first experiment, a total of eight PIV5 vaccine naïve beagles at age of 3 months were divided into two groups, four dogs each infected intranasally (IN) with PIV5 or rPIV5-H3. The dogs were sedated but not anesthetized with Acepromazine (PromAce, Fort Dodge, IA) at a dose of 0.05–0.1 mg/kg intramuscularly for vaccination, and for blood collection and nasal swabs as needed. Blood samples were collected on day 0 (prebleed) and on day 21after infection. Sera were separated from the blood samples and stored at −20°C. Nasal swabs were obtained at 3 and 5 days post infection (dpi). In the second experiment, eight PIV5 vaccinated beagles at age of 5 months were separated into a PBS control group (n = 2) and rPIV5-H3 group (n = 6) immunized via the intranasal route. The dogs were bled on 0 and 21 days following immunization. Nasal swabs were obtained at 3 and 5 dpi. Each IN immunization involved administration of 1 mL of PBS or rPIV5-H3 containing 8×10^7^ plaque forming unit (PFU).

**Figure 6:**
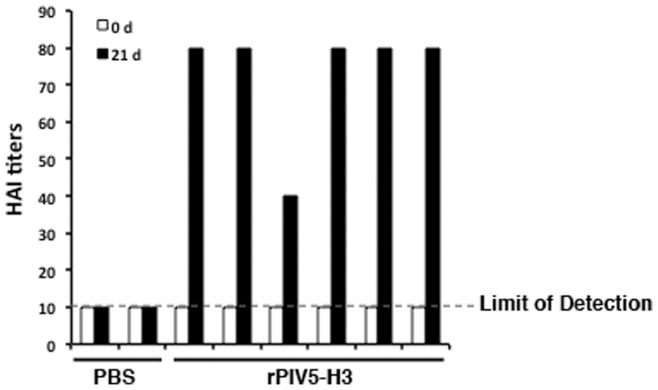
Immune responses in the PIV5-vaccinated dogs inoculated with rPIV5-H3. The dog blood samples were collected at 0 and 21 dpi. Anti-PIV5 and HAI titers were determined using the same approach as in Fig. 3.

### Detecting Virus Using RT-PCR

To obtain nasal swab from dogs, a polyester-tipped flexible aluminum-shafted applicator (Puritan, Maine, USA) was inserted into the naris until resistance was felt at the nasopharynx, then rotated 180 degrees and withdrawn. The swab applicator was removed and absorbent swab was placed into a vial containing 0.5 mL of DMEM with 2% FBS. Vials were stored at −70°C. The specimens were vortexed, and a 140 µL volume was used for total RNA extraction using the QIAamp viral RNA extraction mini kit (Qiagen, CA) according to the manufacturer's instructions. RT-PCR was performed as described before [24]. Briefly, 11 µL of purified RNA template in 30 µL total volume was amplified in a 20 µL reaction volume using Superscript III reverse transcriptase (Invitrogen) to generate virus cDNA. Random primers were used in RT, while gene specific primers P/V-F1 and M-R1annealing to the PIV5 P/V and M gene of the genomic RNA were used in PCR (P/V-F1: 5′-CCAGTGAGGCTCAGCTAATTGACCTC; M-R1: 5′-GGTATTCCCCCGATCCTGTTCGTAG). 5 µL of the cDNAs in 20 µL total volume from RT were used for PCR in a 50 µL reaction volume. Relative levels of viral genome were compared to viral genome levels of PIV5 virus with known titer.

### ELISA

PIV5 or mumps virus-specific antibody titers were determined by Enzyme-linked immunosorbent assays (ELISAs). Ninety-six-well ELISA plates (Thermo Scientific), coated overnight with 100 µl/well of 100 ng purified whole PIV5 virus proteins in PBS (pH 7.4), were blocked first with 0.5% BSA and 0.5% nonfat dry milk in washing solution (KPL) for 1 h and then washed three times with KPL wash solution. Serial dilutions of sera from dogs or humans were prepared in blocking buffer, and incubated for 1 h at room temperature. The plates were washed three times and incubated for 1 h with a 1∶2,000 dilution of an secondary antibody, horseradish peroxidase (HRP)-conjugated goat anti-dog IgG (Santa Cruz, CA) or goat anti-human IgG (KPL, Gaithersburg, MD). The plates were washed three times and developed with SureBlue TMB 1-Component Microwell Peroxidase Substrate (KPL). The development was stopped by the addition of equal volume of 1N HCl, and optical density (OD) was read at 450 nm using an BioTek plate reader. ELISA endpoint titers were defined as the highest serum dilutions at which the mean OD values of duplicate wells were >2-fold above the mean OD value plus 2 standard deviations (SD) for sera.

**Figure 7:**
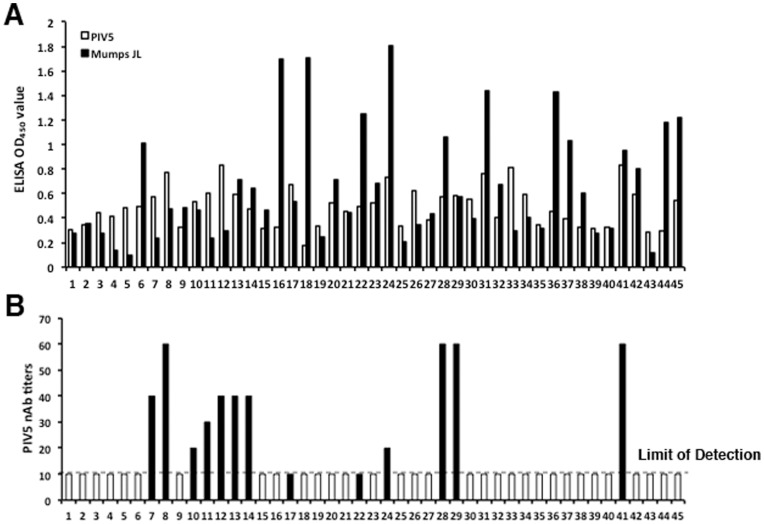
PIV5 antibodies in humans. 45 human serum samples were obtained from 18–50 year old healthy individuals. (A) Comparison of anti-PIV5 and anti-MuV antibody levels. ELISA was performed on plates coated with purified PIV5 or purified MuV with sera serially diluted. PIV5 or Mumps virus specific ELISA OD_450_ values were shown at 320-fold dilution for each human serum sample. (B) Titers of neutralizing antibody against PIV5 in human sera. Data for the antibody titers were the average value of duplicate wells and presented for each human sample. The white column indicates that the PIV5 nAb titer is less than 10, the limit of detection. The black column indicates that the nAb titer is equal to or higher than 10.

### Determining Neutralizing Antibody (nAb) Titers against PIV5

PIV5 neutralizing antibody titers were measured in serum samples by virus neutralizing assay. Sera were serially diluted in 50 µl DMEM containing 2% FBS. 200 TCID_50_ of PIV5 virus was added to diluted sera and incubated for 2 h at 37°C. Serum and virus were added to 96-well microtiter plates containing 90–100% confluent MDBK cells and incubated at 37°C for 3 days. Individual wells were examined by indirect immunofluorescence assay (IFA). Cells were fixed with 3.7% formaldehyde in PBS (pH 7.4) for 10 min, and then permeabilized with 0.1% Triton X-100 plus 1% FBS in PBS for 30 min at room temperature. Fixed cells were incubated for 1 h with primary antibody (mouse anti-PIV5 NP antibody at dilution of 1∶400) at 37°C. FITC-conjugated goat anti-mouse (1∶400 dilution; KPL, Inc.) was used as the secondary antibody. The neutralizing antibody titer was the highest serum dilution completely neutralizing 200 TCID_50_ of PIV5 virus.

### HAI Assay

The hemagglutination inhibition (HAI) assay was performed according to the WHO Manual on Animal Influenza Diagnosis and Surveillance [25]. Briefly, chicken red blood cells (cRBCs) were washed and resuspended to a final concentration of 0.5% in PBS. The influenza A virus (A/Udorn/72, H3N2 subtype) was adjusted to 4 hemagglutination units (HAU) per 25 µl in PBS. In 96-well round-bottom plates, 25 µl of individual RDE-treated serum samples were serially diluted in a two-fold manner. After preparing serial dilution of sera, 25 µl (4 HAU) of the diluted virus was added. The plate was gently mixed and incubated at room temperature for 1 h. Then 50 ul of 0.5% cRBCs were added to each well, gently mixed, and incubated at room temperature for 30–45 minutes. The hemagglutination was scored by tilting the plate at a 45 degree angle. The HAI titer is the reciprocal of the last dilution antiserum that completely inhibits hemagglutination.

### Human Serum Samples

Human blood samples were collected from 45 random volunteers. Participants were healthy, non-pregnant, 18–50 years old and weighed more than 110 pounds. Volunteers signed informed consent forms. The volunteers were anonymous. No personal data were collected. The human subjects protocol (project number: 2012-10769-0, PI: B.R.) was approved by The University of Georgia (UGA) Institutional Review Board. 10 mL venous blood was drawn by venipuncture into a 10 mL tube without anticoagulant in the laboratory of the UGA University Health Center. After clotting, blood samples were centrifuged at 400 g for 5 min. Cell-free supernatants were filtered through 0.22 µm pore size filter units and were used as serum. Serum samples were stored at −80°C.

### Statistical Analysis

In this study, the correlation analysis of antibodies for PIV5 and JL was performed using the Pearson correlation method. OD_450_ readings at 1∶320 was chosen because the readings were in the linear range of sera dilution. The analysis was done with the use of cor.test function in the statistical package R [26]. The statistical significant difference was considered when *p*-value was less than 0.05. The result indicates that there is no significant correlation between PIV5 and JL with Pearson’s r equals 0.06 (*p*-value = 0.6941).

## Results

### Infection of “naïve” Dogs with PIV5 and rPIV5-H3

While our prior studies indicate that rPIV5-H3 is effective in generating immunity in mice against influenza virus, it is not clear whether the same virus can be effective in generating immunity in dogs. We have thus inoculated dogs with rPIV5-H3 via intranasal route, and determined replication of virus in dogs and measured immune responses to the virus. Dogs are routinely vaccinated with vaccines containing live PIV5 at a young age (as early as 3-week old). Through an arrangement with the animal vendor, 8 dogs at 12-week of age without vaccination of live PIV5 were obtained. The titers of PIV5 antibodies in these dogs were determined using ELISA and neutralization assay. All dogs were positive to PIV5 in ELISA (Fig. 1A). However, neutralization antibody (nAb) titers were undetectable (Fig. 1B). The dogs (n = 4) were infected with PIV5 or rPIV5-H3 via intranasal (IN) route. At 3 and 5 days post infection, nasal swabs were taken from infected dogs, and assayed for existence of viruses. While no virus was detected when the swabs were analyzed using plaque assay (Fig. 2), RT-PCR products were detected in 7 of 8 dogs at 3 days post infection (dpi) and very weak RT-PCR signals were detected in 5 of 8 dogs at 5 dpi, suggesting that limited replication of PIV5 in naris of infected dogs at 3 days post-infection and the infection was being cleared at 5 days post-infection. The dogs were bled at 21 days post infection. Increases in anti-PIV5 titers were detected in all dogs, suggesting that the dogs were infected. Measurement of anti-HA titers using HAI assay indicated that all rPIV5-H3 inoculated dogs seroconverted and had HAI titers at average 42.5 (range from 20 to 80) at 3-week post-infection (Fig. 3). No HAI was detected in dogs-inoculated with PIV5.

### Infection of Dogs with Exposure to PIV5 with PIV5-HA

To examine whether dogs with prior exposure with PIV5 can still be vaccinated with recombinant PIV5-based vaccines, we have obtained dogs that were vaccinated against PIV5 multiple times and had anti-PIV5 neutralizing antibodies (Fig. 4). The dogs were infected with rPIV5-H3 via IN route. No virus was detected using plaque assay at 3 and 5 dpi in naris of infected dogs. 1 out 8 dogs tested positive using RT-PCR at 3 dpi (Fig. 5). Dogs were then bled at 3 weeks post-infection. The dogs vaccinated with rPIV5-H3 had HAI titers ranging between 40 to 80 (average 77, 1 at 40 and 5 at 80) (Fig. 6), indicating that rPIV5-H3 vaccination generated immunity against influenza virus (a 4-fold increase of HAI titer or a HAI titer of 40 is considered protective against influenza virus infection). The nAb titers against PIV5 also increased in rPIV5-H3-infected dogs, confirming the infection of the dogs with rPIV5-H3.

### Exposure to PIV5 in Humans

As reported before, anti-PIV5 antibodies were detected in humans [27], [28]. To determine whether the anti-PIV5 in humans is due to cross-reactivity from antibody against closely related paramyxoviruses, we have examined antibody titers of PIV5 and mumps virus in human sera. Mumps virus (MuV) is most closely related to PIV5 as they have same genome structure. Since mumps virus exposure in humans is close to 100 percent due to vaccination and natural infection in the US, we expected to detect anti-mumps virus in all our samples that were collected from 18 to 50-year old in the US. All 45 samples were positive for mumps virus as expected (Fig. 7A). Interestingly, all sera were positive for PIV5 antigen as well on ELISA (Fig. 7A). If reactivity to PIV5 antigen in human sera came from cross-reactivity from anti-mumps virus, titers of anti-PIV5 should correlate to the titers of anti-mumps virus. However, statistical analysis indicated there is no correlation between the titers of anti-MuV in serum and the titers of anti-PIV5 in serum, suggesting that the reactivity of human sera to PIV5 is not due to cross reactivity from mumps virus. Furthermore, we have examined titers of nAb against PIV5 in human sera and have detected anti-PIV5 nAb in 13 out of 45 samples (about 29 percent) (Fig. 7B).

## Discussion

Since the discovery of PIV5, many diseases in humans were associated with PIV5, which all were ultimately proved false. In retrospect, several possible explanations exist for why PIV5 may have been linked to these diseases. One is based on the conditions used for virus isolation in the human studies, i.e. the labs used monkey cell lines which can be persistently infected with PIV5, and these cells often show no detectable cytopathic effects [6], [7]. Another possibility is antigen cross-reactivity of PIV5 to ubiquitous paramyxoviruses such as mumps virus, which are closely related to PIV5 and have almost 100 percent exposure in human population [29], [30], [31]. In this work, we have examined exposure of PIV5 in human populations. We have found anti-PIV5 antibodies in human sera. Interestingly, we did not detect a correlation between titers of anti-mumps virus and anti-PIV5, suggesting that PIV5-positve humans, at least some of them, might have been exposed to PIV5. About 29% of the human serum samples had neutralizing antibodies against PIV5. Some of which did not have robust antibody against mumps virus (Fig. 7), suggesting that at least some humans have been exposed to PIV5 separately from mumps virus. We propose that close contact between dogs and humans may be a contributing factor in exposure of humans to PIV5 from dogs. Dogs are vaccinated intranasally and often sneeze during the vaccination, exposing veterinary workers and owners as well. In addition, PIV5 was detected in naïve dogs at 3 dpi using RT-PCR, suggesting that it is possible that vaccinated dogs may shed virus after vaccination, resulting in humans being exposed to the virus. This hypothesis is consistent with the wide spread use of kennel cough vaccines that contain live PIV5 and that approximately 40% of the US population are dog owners.

It is encouraging that PIV5 antibody is detected in a large percentage of the US population without causing clinical disease, which suggests that PIV5 is safe in human populations. However, because a large percentage of the US population may have been exposed to PIV5, it raises the question whether PIV5 will be an effective vector for vaccine development in humans. The very same problem of prior exposure of vector has created a major obstacle for using adenovirus-based vector for vaccine development. In this work, we have found that recombinant PIV5 expressing HA was immunogenic in dogs with pre-existing immunity against PIV5, indicating that PIV5-based vaccine vector can overcome pre-existing immunity. The results were consistent with a previous report that in mice neutralizing antibodies against PIV5 do not prevent PIV5 infection [32]. The dog's ability to clear a PIV5 infection remains undetermined. In mice, it is thought that cell-mediated immune responses play a critical role in clearing PIV5 infection [32]. Since PIV5 has self-limiting replication in dogs, it is likely that cell-mediated immunity plays a critical role in clearing infection as well. Because it takes time for cell-mediated immunity to respond and be effective, this time period provides a window of opportunity for PIV5-based live vaccine to replicate and generate a robust immune response. This is consistent with the observations that PIV5 infects all kinds of cells, including primary cells [1], [10], [20] [36].

The nAb titers against PIV5 in vaccinated dogs were higher than the “naïve” dogs and were as high as 300 (Fig. 4B). All dogs with nAb against PIV5 seroconverted after a single dose IN inoculation of rPIV5-H3, and the titers of anti-H3 antibody had no correlation to the nAb titters against PIV5, further confirming that nAbs of PIV5 had no predictive value in determining immune responses to a PIV5-based vaccine in dogs. The highest titer of nAb against PIV5 in humans is 60, lower than the titers of nAb against PIV5 in dogs. Thus, we hypothesize that neutralizing antibody against PIV5 in humans will not prevent PIV5-based vaccine candidates from generating protective immunity. The serum samples used in this study came from de-linked sources, thus, very limited information is known about these donors except they were from 18 to 50 years old residents of the USA, who were likely vaccinated with mumps virus. It will be interesting to determine whether there is a relationship between age and/or contact with dogs and titers of anti-PIV5 nAb.

Outbreaks of canine influenza A virus subtype H3 have occurred in canine populations [33]–[35]. The fact that rPIV5-H3 seroconverted dogs and generated immunity that is considered protective suggests that a recombinant PIV5 expressing H3 may be an effective vaccine against canine influenza virus. Furthermore, these results suggest that PIV5 can be a novel vector for expressing other antigens for vaccine development for dogs, other animals and humans.
